# Guest Perceptions of Physical Activity Point-of-Decision Prompts at a Conservatory with Botanical Gardens

**DOI:** 10.3390/ijerph16122074

**Published:** 2019-06-12

**Authors:** Corinne Natale, Mary Kathryn Poole, Emily A. Kalnicky, Sharon E. Taverno Ross

**Affiliations:** 1 Department of Physical Therapy, MGH Institute of Health Professions, Boston, MA 02129, USA; cnatale9@gmail.com; 2 Department of Nutrition, Harvard T.H. Chan School of Public Health, Boston, MA 02115, USA; mkp984@g.harvard.edu; 3 Minnesota Zoological Gardens, Apple Valley, MN 55124, USA; emily.kalnicky@state.mn.us; 4 Department of Health and Physical Activity, University of Pittsburgh, Pittsburgh, PA 15260, USA

**Keywords:** physical activity promotion, botanical garden, children, adult, public health

## Abstract

Point-of-decision prompts are cost-effective strategies to promote physical activity in public spaces. This study explored how adult and child guests of a conservatory with botanical gardens perceived point-of-decision prompts that aimed to promote physical activity. Seven point-of-decision prompts were developed and displayed throughout the conservatory. Adult guests (*n* = 140) were invited to complete a voluntary and anonymous survey to assess awareness of point-of-decision prompts, adult–child interactions, and physical activity engagement. Descriptive statistics were calculated using SPSS version 23. Sixty-one percent of guests (*n* = 86) who responded to the survey noticed the point-of-decision prompts. Over 65% (*n* = 56) of those guests completed at least one of the physical activities, and 53% (*n* = 46) completed one to three. Of guests attending with (a) child(ren) (*n* = 17) who completed the survey, over half (*n* = 9) engaged in at least one physical activity together. In sum, the point-of-decision prompts were noticed by some guests in this public space. More research is needed to determine whether point-of-decision prompts are able to lead to sustainable behavior change.

## 1. Introduction

Evidence-based recommendations, such as the U.S. National Physical Activity Plan, have been established to increase public engagement in physical activity [1]. The Plan is a comprehensive set of policies, programs, and initiatives that seek to promote physical activity in all individuals in the United States, both adults and children. The Community Recreation, Fitness, and Parks sector of the Plan highlights the many facilities and services available to encourage more physical activity, including indoor and outdoor public parks and recreation facilities. In spite of such resources, most adults and children in the United States are not meeting daily recommendations for physical activity [2,3]. Therefore, there is a need to strategize ways to increase individuals’ physical activity, accumulated throughout the day, to improve the health of the nation.

The Centers for Disease Control (CDC) Community Preventive Services Task Force recommends point-of-decision prompts as one cost-effective and evidence-based strategy to increase physical activity [4]. A review of effective physical activity interventions confirmed that point-of-decision prompts were the most cost-effective strategy due to their overall low-cost and the large populations that they reach [5]. Point-of-decision prompts are motivational signs placed strategically in public areas, a generally open and accessible space, to promote physical activity [4]. Examples include point-of-decision signs placed near staircases to encourage stair-use or in park spaces to encourage people to be active. In a review study of point-of-decision prompts and their impact on stair-use, Bellicha et al. [6] included 50 articles: 25 intervention studies in worksites and 35 intervention studies in public settings. They found an increase in stair-use during the intervention period in 64% of the studies in worksites and 76% of studies in public settings, supporting the effectiveness of this low-touch strategy.

Fewer studies have used point-of-decision prompts to encourage physical activity in public spaces, outside of stair-use. One study by Kaczynski et al. [7] examined the effectiveness of point-of-decision prompts in parks on intention to be active. In this study, participants were randomly exposed to either a photo of a park with a message on a sign, or an identical photo of the park with no prompt in the photo. The sign in the picture was green and read: “Take a walk around the park! Doctors recommend that being active just 30 minutes per day can help you maintain a healthy weight and ward off many diseases.” The findings indicated that individuals exposed to the sign with messaging reported significantly greater intentions to be active. Such findings suggest that calling attention to the benefit of physical activity in public areas may be effective at encouraging physical activity behaviors. However, physical activity behavior was not assessed in this study.

Previous studies have focused exclusively on point-of-decision prompts to increase physical activity in adult populations, primarily through stair-use. There is a dearth of information surrounding the effectiveness of point-of-decision prompts to increase physical activity in children or through adult–child interaction. As “gatekeepers” and “agents of change” in children’s physical activity behaviors, it is logical that interventions to increase child physical activity would also engage parents [8,9]. Alternatively, in the school setting, a practical tool was created to outline evidence-based design guidelines for school architecture to promote physical activity [10]. In the signage and wayfinding domain, the authors cited substantial evidence for the use of point-of-decision prompts for stairs and other school-based physical activity opportunities. Also included in this recommendation was the incorporation of physical activity signage that is educational, promotes its benefits, but is also age-appropriate and fun. As such, point-of-decision prompts may be a promising way to increase child physical activity levels, potentially through co-activity with an adult.

To our knowledge, no previous studies have examined the utility of point-of-decision prompts to encourage both adults and children to engage in physical activity in a public space. Previous studies have looked exclusively at adults and it is unknown how children would respond to such signage. Further, few studies have used point-of-decision prompts in public spaces, and no studies have looked specifically at physical activity prompts in a conservatory with botanical gardens. Therefore, the purpose of this study was to describe the perceptions of and reactions to point-of-decision prompts to encourage physical activity among adult and child guests of a conservatory with botanical gardens.

## 2. Materials and Methods

### 2.1. Subjects/Setting

This study was conducted at Phipps Conservatory and Botanical Gardens (Phipps) in Pittsburgh, PA, USA. Phipps welcomes over 400,000 guests a year to view its seasonal displays and to participate in an array of educational programs [11]. To support its goal of being a premier organization for children, connecting them to nature, promoting healthy and sustainable lifestyles, Phipps launched a healthy lifestyle initiative in 2011—*Let’s Move Pittsburgh* [12]. Modeled after former First Lady Michelle Obama’s *Let’s Move!* initiative, Let’s Move Pittsburgh is a program that provides children and their caregivers with the knowledge, tools, and support needed to make nutritious food choices and lead active lifestyles through educational materials and activities within the conservatory, as well as in early childcare centers, schools, and out-of-school sites.

### 2.2. Intervention Development

In 2016, the Let’s Move Pittsburgh department and the Phipps Research Institute for Biophilia and Science Engagement collaborated with the University of Pittsburgh to develop strategies for encouraging guests to be physically active during their visit to Phipps. Given the extensive literature supporting point-of-decision prompts as a cost-effective strategy to promote physical activity [5], the team selected this strategy to promote physical activity in all guests - adults and children.

**Figure 1:**
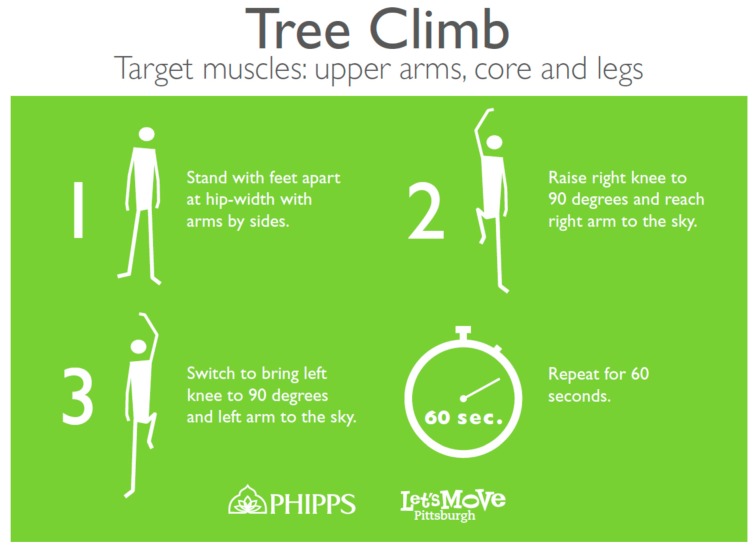
Physical activity point-of-decision prompt sign. Each physical activity prompt displayed the name of the activity, a description of the physical activity, the target muscles of the physical activity, and an instructional picture of the activity. The picture and text description of the physical activity were printed in white on the light-green background of the signs. This sign highlights the “Tree Climb” activity that targeted the guests’ upper arms, core, and legs.

Over three months, the team planned and developed the sign design adapted from those used in the adult-focused Kaczynski et al. [7] study, and the graphics of the Empower Me indoor fitness trail for children from the Alliance for a Healthier Generation [13]. Seven point-of-decision prompts were designed to be engaging, encourage activities that elevated heart rate, and could be easily understood by guests of all ages, reading levels, and fitness levels (Supplementary Materials). Picture and text descriptions of the physical activities were printed in white on a light-green background, as these colors are associated positively with health and wellness [14] (Figure 1).

### 2.3. Intervention Description

**Figure 2:**
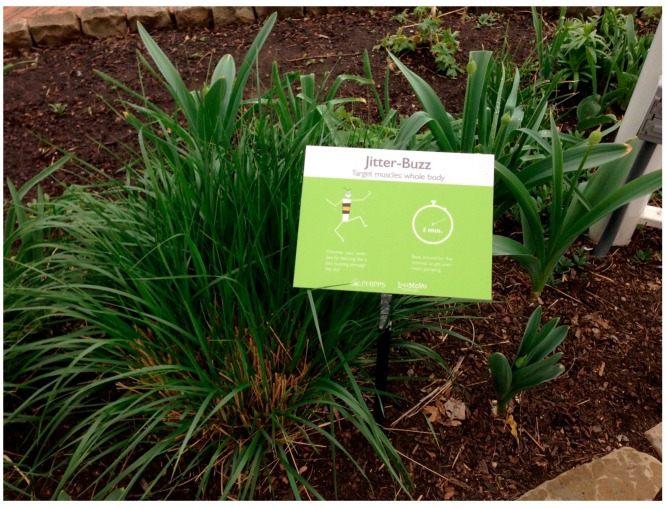
Placement of a point-of-decision prompt in the conservatory with botanical gardens. In this example, the sign was placed on the ground in a plant, in the Discovery Garden gazebo. This “Jitter Buzz” activity targeted whole body movement in guests. Instructions included: (1) Discover your inner bee by dancing like a bee buzzing through the sky! (2) Buzz around for 5 minutes to get your heart pumping.

The seven point-of-decision prompts were designed to correspond to the theme of the permanent horticultural displays in each room or area of the conservatory (e.g., Jitter Buzz in the Discovery Garden gazebo) (see Figure 2). The physical activities were low-touch, meaning that they required no equipment, and could be completed without getting down on the floor. To promote guest awareness and engagement, upon arrival, guests with children under 12 were offered a scavenger hunt handout at the conservatory entrance. The handout included a checklist of the physical activity signs, including the name and room location. Children who completed the checklist were awarded a small prize (e.g., a sticker) when exiting the conservatory.

### 2.4. Data Collection

A trained research assistant stationed at the exit of the conservatory invited exiting adult guests to complete a voluntary and anonymous survey based on their experience. The research assistant stood at the table where there were paper copies of the survey for guests to complete and a drop-box in which to submit the surveys. These surveys were collected on four separate Saturdays during the months of May through August 2016. All study procedures were approved by the Institutional Review Board at the University of Pittsburgh with exempt status (PRO16090597).

### 2.5. Measures

The survey included eight questions about guest awareness and engagement with the point-of-decision prompts. The first question asked guests to indicate whether they noticed any of the point-of-decision prompts displayed throughout the conservatory. Those guests who reported noticing the point-of-decision prompts during their visit were instructed to answer an additional three questions. Specifically, guests provided information about the number of physical activity signs for which they completed the physical activity using a four-point scale: None (0), some (1–3), most (4–6) or all (7). Next, the guests rated the effectiveness of the physical activity signs (i.e., did the physical activity signs make you want to be physically active?) on a four-point scale (very ineffective, somewhat ineffective, somewhat effective, or highly effective). Finally, guests reported how likely they would be to try the physical activities at home (very unlikely, somewhat unlikely, somewhat likely, or highly likely).

Guests who had (a) child(ren) with them during their visit were asked to answer four additional questions. Specifically, guests reported the number of children with them, as well as how often they: a) needed to read the description of the target physical activity to the child(ren), b) how often the child(ren) needed help interpreting the activity described on the physical activity signs, and c) how often the adult guests participated in the activity with the child(ren). Response options included: none of the time, some of the time, most of the time, or not applicable.

### 2.6. Statistical Analyses

Descriptive statistics were used to summarize the survey items using IBM SPSS Statistics for Windows, version 23 (IBM Corp., Armonk, NY, USA). Specifically, frequencies and percentages were calculated for each response option for all eight survey questions. One participant mistakenly answered three of the final questions that pertained to guests who had child(ren) with them; as such, the participant’s responses to these questions were removed from the analyses.

## 3. Results

One hundred and forty guests completed the exit survey. Sixty-one percent (*n* = 86) reported noticing at least one of the physical activity signs (Table 1). Of those guests who noticed the signs, more than 65% (*n* = 56) reported completing at least one of the physical activities, with 53% (*n* = 46) completing one to three physical activities, 5% (*n* = 4) completing four to six physical activities, and 7% (*n* = 6) completing all seven of the physical activities (Table 1).

When asked about the physical activity signs’ overall ability to encourage/prompt physical activity, 60% (*n* = 52) of guests who completed the survey rated the physical activity signs as somewhat or highly effective. Nearly 40% of these guests (*n* = 34) said they were somewhat likely or very likely to try the physical activities at home, while the remaining 60% (*n* = 52) reported they were unlikely to try the physical activities at home (Table 1).

Only 20% (*n* = 17) of guests who reported noticing the physical activity signs also reported that they were accompanied by at least one child during their visit to the conservatory that day (Table 1). Over half (*n* = 9) of these guests reported participating in the physical activities along with the child(ren) for at least one of the point-of-decision prompts. Furthermore, 41% (*n* = 7) reported helping the child(ren) interpret the movements on the physical activity sign, and 47% (*n* = 8) reported reading at least one physical activity sign aloud to the child(ren).

## 4. Discussion

This study found that over half of the guests to a conservatory with botanical gardens who completed a voluntary survey noticed the point-of-decision prompts to encourage physical activity in the space. Of those who noticed the signs, more than half also reported completing at least one of the physical activities during their visit. The physical activity signs were helpful in promoting physical activity in some, but not all, participants. Similarly, one previous study found that those participants exposed to the point-of-decision prompts reported significantly greater intentions to be active than those who viewed a photo without a prompt [7].

We did not collect demographic data and therefore are unable to determine if responses to the point-of-decision signs varied by guest characteristics. It would be helpful to understand more about those individuals who noticed the signs, including specific demographic characteristics or other factors (e.g., physical activity self-efficacy), that may have influenced their awareness and subsequent engagement. A review of previous studies reported that point-of-decision prompts for stair-use were equally effective across subpopulations regardless of (adult) age categories, gender, or race/ethnicity [15]. Interestingly, in the Kaczynski study with park-based point-of-decision prompts [7], the effect was stronger for women than men. Next steps in the current study could include focus groups with the high awareness/high engagement group to yield important information about why they noticed the signs and which signs had a bigger impact on them. Understanding more about this high-responder group could assist researchers to maximize point-of-decision prompt effectiveness in a conservatory with botanical gardens.

While seven physical activity signs were posted throughout the conservatory with botanical gardens, few guests reported completing the movements on all of the physical activity signs. Despite noticing the signs/prompts, over one-third of guests did not participate in any of the physical activities. Additionally, less than half of guests indicated that they were likely to try the activities at home. There is an increased awareness of the need to promote physical activity in the U.S. population, which is overwhelmingly sedentary [16]. Various recommendations have been put forth to engage individuals in physical activity while they are in free and accessible areas [1]. While point-of-decision prompts are evidence-based strategies to promote increased physical activity in individuals, it is likely the total additional minutes of physical activity over the day would be few. However, in combination with other physical activity breaks throughout the day, point-of-decision prompts could help individuals meet their daily physical activity recommendations and promote a culture of activity in public spaces.

This study is one of the first to explore the effectiveness of point-of-decision prompts for both adults and children in public spaces. There is a shortage of research examining whether point-of-decision prompts may influence engagement in physical activity outside of these initial public spaces (e.g., at home). It is unclear whether the point-of-decision prompts would encourage more physical activity outside of the public space, or perhaps would lead individuals to compensate with decreased physical activity throughout the day [17]. Conducting follow-up interviews with guests regarding their behavior after leaving the conservatory with botanical gardens would provide a deeper understanding of participant motivation, intentions, and physical activity behaviors. Further, a follow-up study with repeat botanical garden visitors to determine if they follow the activity prompts on multiple visits, or only on their first visit, could help to determine the sustained interest in physical activity resulting from the prompts.

Physical activity sign placement and limited visibility may have contributed to low levels of guest awareness and engagement. For example, one researcher in the study noted that some of the signs had low visibility due to the horticultural displays in the rooms (e.g., placed behind a large plant). Moreover, it is possible that the effectiveness of the point-of-decision prompts may differ by type of setting as well as location within a setting. It is important to adapt signs to the specific setting and target population [15], which was a main goal in our intervention design. The fact that many of the guests who completed the survey were visiting the conservatory without children may have led to lower participation due to social norms or embarrassment of adults participating in random physical activity through prompts in a public space. It is also possible that the signs were assumed to be more of a child-focused activity, since the adults without child(ren) did not receive a copy of the scavenger hunt. Studies that provide insight into these differences could inform key recommendations for how to integrate physical activity signs effectively within public spaces. Future research should also explore whether additional materials, including handouts, may influence guest awareness.

This is an observational study and cannot provide inferences about the effectiveness of point-of-decision signs in increasing physical activity. Because exact attendance was not recorded for each day, and the survey was voluntary, it is unclear what proportion of the total daily guests actually engaged in the point-of-decision signs. It is possible that those guests who completed the survey voluntarily were more invested in the topic or the conservatory, which could have biased the results; however, we did not collect information about how many guests were approached or how many declined to complete the survey. A larger sample of adults with children would also help to better understand the degree to which the point-of-decision prompts facilitated adult–child physical activity interaction. Another limitation was that there was not a mandatory pathway through which the guests moved about the conservatory, and they may not have entered every room or area. It is possible that physical activity sign awareness would have increased if guests were directed to move through each room consecutively or in a uniform direction. Further, improved visibility of the signs, through the size or display location, may have also increased guest awareness.

## 5. Conclusions

In line with the CDC’s Task Force on Community Preventive Services, the results of this study suggest that inclusion of point-of-decision prompts in a conservatory with botanical gardens moderately engaged guests in physical activity in this public space. More research is needed to understand the effectiveness of such point-of-decision prompts for increasing guests’ physical activity. Though increases in physical activity would be small [5], in combination with similar efforts in other public settings, physical activity breaks through point-of-decision prompts could contribute to helping individuals meet daily physical activity guidelines. Such efforts would also align with the U.S. National Physical Activity Plan Alliance recommendations for increasing physical activity within the Community Recreation, Fitness, and Parks sector of the country [1]. Further, given the potential of physical activity signs to engage a large number of individuals in additional small physical activity breaks throughout the day, efforts should focus on understanding ways to maximize the effectiveness of these low-touch and low-cost methods for broad public implementation and practice in public spaces beyond botanical gardens.

## Figures and Tables

**Table 1:** Guest awareness of and participation with point-of-decision prompts, as well as adult–child interactions at a Conservatory with Botanical Gardens.

Survey Item	Response Options	*n* (%)
Did you notice the new physical activity signs during your visit to Phipps today?	Yes No	86 (61%) 54 (39%)
**Of those guests who noticed the signs**		
Of the seven physical activity signs posted throughout Phipps, for how many signs did you do the activity?	None (0) Some (1–3) Most (4–6) All (7)	30 (35%) 46 (53%) 4 (5%) 6 (7%)
How effective were the physical activity signs in encouraging you to complete the movements (i.e., did they make you want to move)?	Very or Somewhat ineffective Somewhat or Highly effective No Answer	29 (34%) 52 (60%) 5 (6%)
How likely are you to try the physical activities listed on the signs at home?	Very or Somewhat unlikely Somewhat or Very likely	52 (60%) 34 (40%)
How many child(ren) are with you today?	1 2 3+	6 (35%) 10 (59%) 1 (6%)
**Of those guests who reported visiting with children**		
How often did you need to read the physical activity signs to your child(ren)?	None of the Time Some of the Time Most of the Time Not Applicable ^1^	7 (41%) 2 (12%) 6 (35%) 2 (12%)
How often did your child(ren) need help interpreting the movements listed on the physical activity signs?	None of the Time Some of the Time Most of the Time	10 (59%) 3 (18%) 4 (23%)
How often did you participate in the movements listed on the physical activity signs with your child(ren)?	None of the Time Some of the Time Most of the Time	8 (47%) 2 (12%) 7 (41%)

^1^ The ‘Not applicable’ response for the question ‘How often did you need to read the physical activity signs to your child(ren)?’ likely indicated that the guest attended with a child but did not engage in any physical activity as prompted by the sign.

## Supplementary Materials

The following are available online at https://www.mdpi.com/1660-4601/16/12/2074/s1, Figure S1: Physical Activity Point-of-Decision Prompt Signs (Complete).
