# Supplementary material for: Guest Perceptions of Physical Activity Point-of-Decision Prompts at a Conservatory with Botanical Gardens
Source: Int J Environ Res Public Health. 2019 Jun 12;16(12):2074. doi: 10.3390/ijerph16122074 (PMC6616916; doi:10.3390/ijerph16122074)
Supplement: Supplementary file 1 [file ijerph-16-02074-s001.zip › online-supp.pdf]

Tree Pose

Target muscles: shoulders, back and legs

1

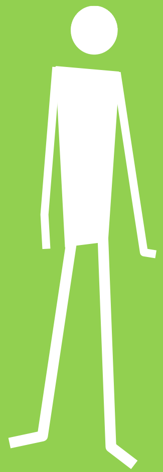

Stand with feet apart at hip-width keeping toes facing forward.

2

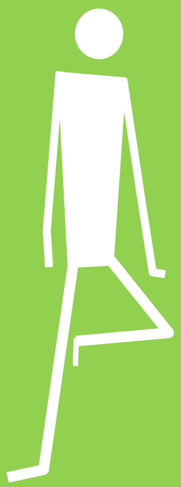

Bend left knee and place sole of left foot on the inside of right calf or thigh with toes pointing downward.

3

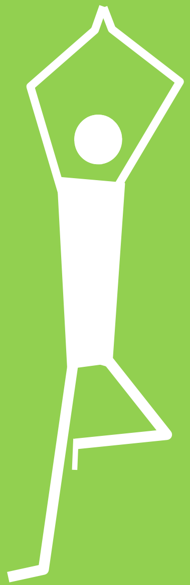

Slowly raise both arms above the head and place palms together, fingers upward.

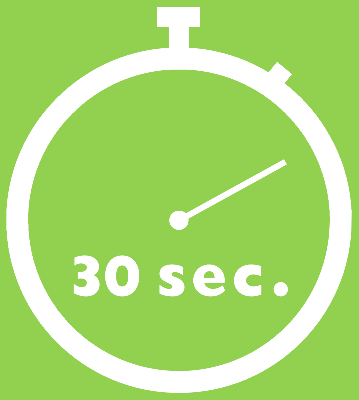

Balance for 30 seconds, and repeat bringing right foot to left leg.

PHIPPS

Let's Move Pittsburgh

What's Under the Rock?

Target muscles: shoulders, back and legs

1

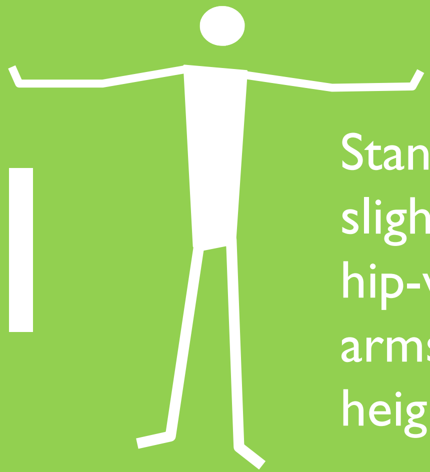

Stand with feet apart slightly wider than hip-width and raise arms to shoulder height.

2

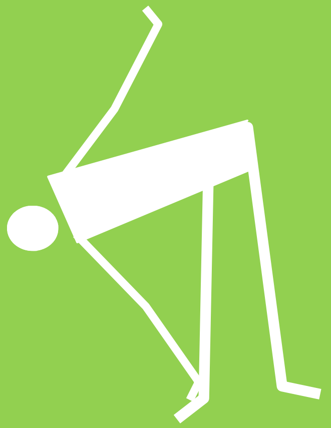

Bend and touch your left foot with your right hand.

3

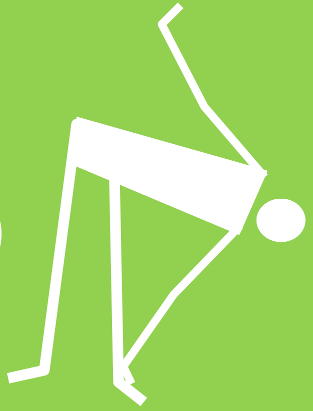

Repeat, touching your right foot with your left hand.

2 x 10

One set is touching both feet. Complete two sets of 10.

PHIPPS

Let's Move Pittsburgh

Tree Climb

Target muscles: upper arms, core and legs

1

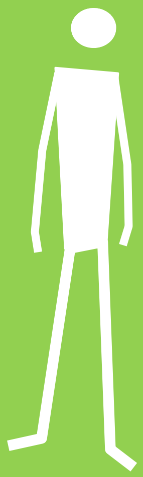

Stand with feet apart at hip-width with arms by sides.

2

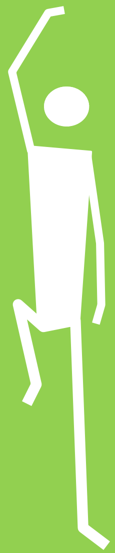

Raise right knee to 90 degrees and reach right arm to the sky.

3

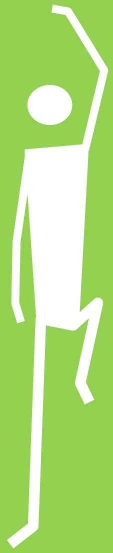

Switch to bring left knee to 90 degrees and left arm to the sky.

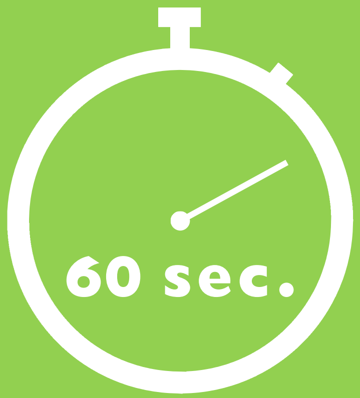

Repeat for 60 seconds.

PHIPPS

Let's Move Pittsburgh

Yoga Sunrise

Target muscles: full body stretch

1 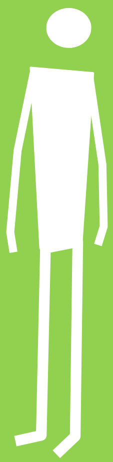 Stand with feet together.

2 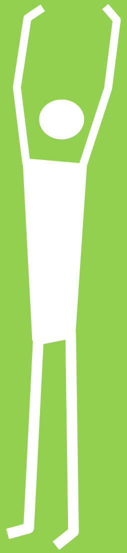 Reach arms to the sky.

3 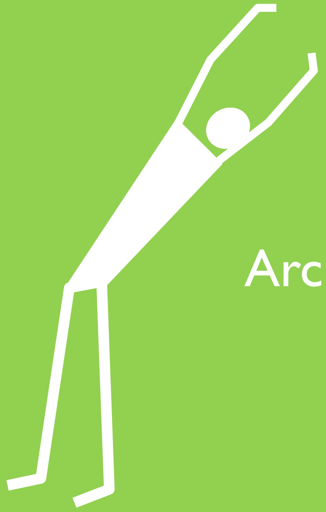 Arch back.

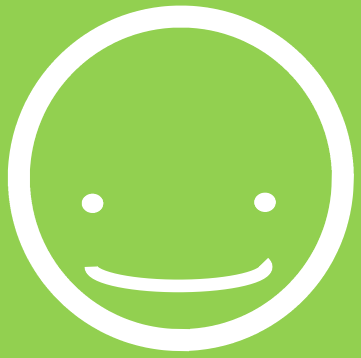 Repeat until refreshed.

PHIPPS

Let's Move Pittsburgh

Butterfly Figure Eights

Target muscles: arms

1

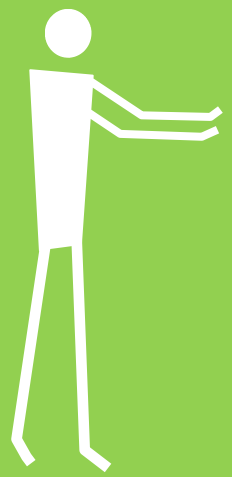

Extend both arms in front of you.

2

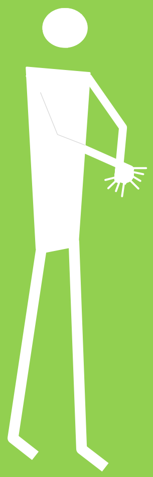

Make a butterfly with hands by crossing arms and clasping thumbs while keeping other fingers extended.

3

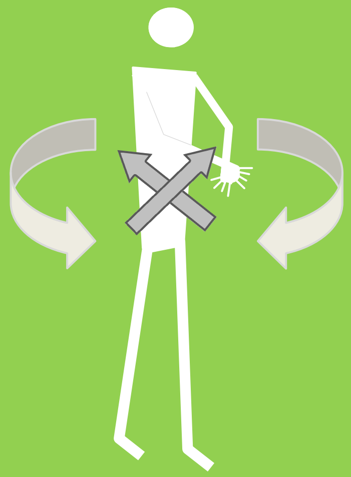

Trace large figure eights in the air while fluttering fingers.

x 20

Reverse figure eight and repeat 20 times.

PHIPPS

Let's Move Pittsburgh

Jitter-Buzz

Target muscles: whole body

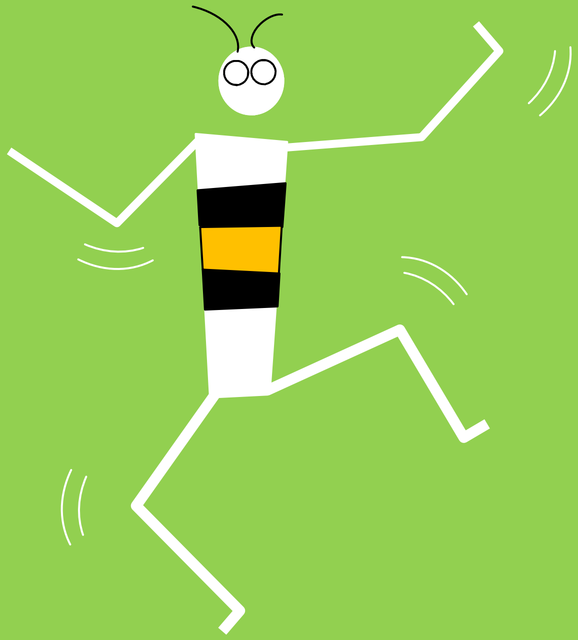

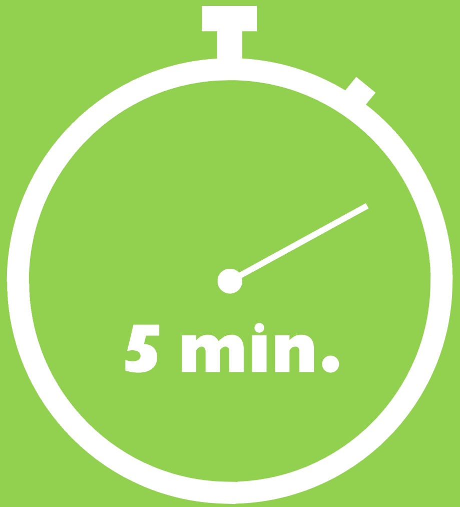

Discover your inner bee by dancing like a bee buzzing through the sky!

Buzz around for five minutes to get your heart pumping.

PHIPPS

Let's Move Pittsburgh

Skip Around the World

Target muscles: Quadriceps, hamstrings and abs

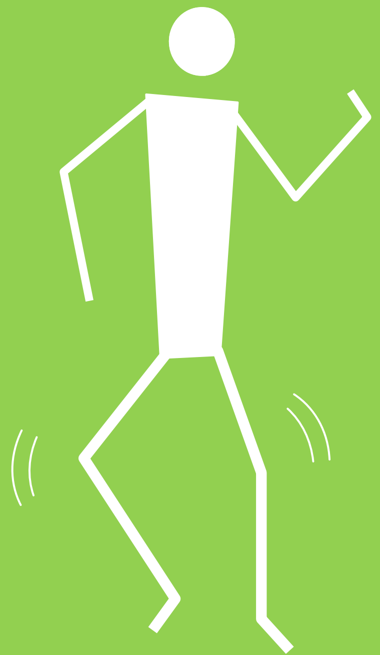

Step from right foot to left foot with a light bounce.

10 x

Circle the large round sign 10 times.

PHIPPS Let's Move Pittsburgh
